# Deconvolution of multiple infections in *Plasmodium falciparum* from high throughput sequencing data

**DOI:** 10.1093/bioinformatics/btx530

**Published:** 2017-08-22

**Authors:** Sha Joe Zhu, Jacob Almagro-Garcia, Gil McVean

**Affiliations:** 1 Wellcome Trust Centre for Human Genetics, University of Oxford, Oxford, UK; 2 Big Data Institute, Li Ka Shing Centre for Health Information and Discovery, University of Oxford, Oxford, UK; 3 Medical Research Council (MRC) Centre for Genomics and Global Health, University of Oxford, Oxford, UK; 4 Wellcome Trust Sanger Institute, Hinxton, UK

## Abstract

**Motivation:**

The presence of multiple infecting strains of the malarial parasite *Plasmodium falciparum* affects key phenotypic traits, including drug resistance and risk of severe disease. Advances in protocols and sequencing technology have made it possible to obtain high-coverage genome-wide sequencing data from blood samples and blood spots taken in the field. However, analyzing and interpreting such data is challenging because of the high rate of multiple infections present.

**Results:**

We have developed a statistical method and implementation for deconvolving multiple genome sequences present in an individual with mixed infections. The software package *DEploid* uses haplotype structure within a reference panel of clonal isolates as a prior for haplotypes present in a given sample. It estimates the number of strains, their relative proportions and the haplotypes presented in a sample, allowing researchers to study multiple infection in malaria with an unprecedented level of detail.

**Availability and implementation:**

The open source implementation *DEploid* is freely available at https://github.com/mcveanlab/DEploid under the conditions of the GPLv3 license. An R version is available at https://github.com/mcveanlab/DEploid-r.

**Supplementary information:**

Supplementary data are available at *Bioinformatics* online.

## 1 Introduction

Malaria remains one of the top global health problems. The majority of malaria related deaths are caused by the *Plasmodium falciparum* parasite (WHO, 2016), transmitted by mosquitoes of the genus *Anopheles*. Patients are often infected with more than one distinct parasite strain (termed mixed infection, multiple infection, or complexity of infection), due to bites from multiple mosquitoes, mosquitoes carrying multiple genetic types or a combination of both. Mixed infections can lead to competition among co-existing strains and may influence disease development (de Roode *et al.*, 2005), transmission rates (Arnot, 1998) and the spread of drug resistance (de Roode *et al.*, 2004). In addition, within-host evolution can lead to the presence of more than one genetically and phenotypically distinct strains (Bell *et al.*, 2006).

The presence of multiple strains of *P.falciparum* makes fine scale analysis of genetic variation challenging, since genetic differences between strains of this haploid organism will appear as heterozygous loci. Such mixed calls confound methods that exploit haplotype data to detect, among other phenomena, the occurrence of natural selection or recent demographic events (Harris and Nielsen, 2013; Lawson *et al.*, 2012; Mathieson and McVean, 2014; Sabeti *et al.*, 2002). In light of these difficulties, researchers usually focus on clonal infections or resort to heuristic methods for resolving heterozygous genotypes. The former approach discards valuable information regarding genetic diversity and relatedness, whereas the latter tends to create chimeric haplotypes that are not suitable for analysis, unless mixed calls are very sparse.

In comparison to the problem of phasing haplotypes within diploid organisms, deconvolving the strains of a multiple infection differs because of uncertainty in the number of strains present and their relative proportions. Consequently, existing tools for phasing diploid organisms, such as BEAGLE (Browning and Browning, 2007), IMPUTE2 (Howie *et al.*, 2009) and SHAPEIT (Delaneau *et al.*, 2012; O’Connell *et al.*, 2014), are not appropriate. Galinsky *et al.* (2015), O’Brien *et al.* (2016) and Chang *et al.* (2017) have attempted to address the multiple infection problem by inferring the number and proportions of strains from allele frequencies within samples. However, since they do not infer haplotypes, these approaches have limited applicability.

As part of the Pf3k project (www.malariagen.net/data/pf3k-5), an effort to map the genetic diversity of *P.falciparum* at global scale, we have developed algorithms and a software package implementation DEploid, for deconvolving multiple infections. The program estimates the number of different genetic types present in the isolate, the proportion or abundance of each strain and their sequences (i.e. haplotypes). To our knowledge, DEploid is the first package able to deconvolute strain haplotypes and provides a unique opportunity for researchers to study the epidemiology of *P.falciparum*.

## 2 Materials and methods

The following sections provide details on the statistical model used for inference and its implementation, with additional detail being provided in the Supplementary Material. Readers wishing to skip technical details can move directly to the section on validation and performance.

### 2.1 Notations

We first introduce our notation (see Table 1). Our data, *D*, are the allele read counts of sample *j* at a given site *i*, denoted as $r_{j,i}$ and $a_{j,i}$ for reference (REF) and alternative (ALT) alleles respectively. These are assigned values of 0 and 1, resepctively. Here we consider only biallelic loci, though future extension to include multi-allelic sites is simple. The empirical allele frequencies within a sample (WSAF) $p_{j,i}$ and at population level (PLAF) *f_i_* are calculated by $\frac{a_{j,i}}{a_{j,i}+r_{j,i}}$ and $\frac{\sum_{j}a_{j,i}}{\sum_{j}a_{j,i}+\sum_{j}r_{j,i}}$ respectively. Since all data in this section refers to the same sample, we drop the subscript *j* from now on.

**Table 1.** Notation used in this article

Symbol	Notation
*i*	Marker index
*j*	Sample index
*r*	Read count for reference allele
*a*	Read count for alternative allele
*f*	Population level allele frequency (PLAF)
*n*	Number of strains within sample
*l*	Sequence length
**w**	Proportions of strains
**x**	Log titer of strains
$h_{i}$	Allelic states of *n* parasite strains at site *i*
$h_{k,i}$	Allelic state of parasite strain *k* at site *i*
*p*	Observed within sample allele frequency (WSAF)
*q*	Unadjusted expected WSAF
π	Adjusted expected WSAF
Ξ	Reference panel
$\xi_{k,i}$	Allelic state of reference panel strain *k* at site *i*
*G*	Scaling factor used for genetic map
*e*	Probability of read error

### 2.2 Model

We describe the mixed infection problem by considering the number of strains, *n*, the relative abundance of each strain, **w**, and their allelic states, **h**. Similar to O’Brien *et al.* (2016), we use a Bayesian approach and define the posterior probabilities of *n*, **w** and **h** given a reference panel, Ξ, and the read error rate, *e*, as:

(1) P(n,w,h,| Ξ,e,D)∝L(n,w,h,| Ξ,e,D)×P(n,w,h).

We assume a prior in which the haplotypes of the *n* strains are independent of each other and dependent only on the reference panel. Therefore, the joint prior can be written as:

(2) $$P(n,w,h)=P(n)\times P(w|n)\times \prod_{k=1}^{n}P(h_{k}|\;\Xi ).$$

The following sections describe details of the model and the approach to inference.

#### *2.2.1* Likelihood function

Let $w=[w_{1},\ldots ,w_{n}]$ and $h_{i}=[h_{1,i},\ldots ,h_{n,i}]$ denote the proportions and alleic states of the *n* parasite strains at site *i*. We use O’Brien *et al.* (2016)’s expression for the expected WSAF at site *i*, *q_i_*, as:

(3) $$q_{i}=(w\cdot h_{i})=\sum_{k=1}^{n}w_{k}\cdot h_{k,i}.$$

The data, which can be summarized by the reference and alternative allele read counts at each site, is modelled through a beta-binomial distribution given the expected WSAF. We model the data at distinct segregating sites as independent. Thus the likelihood function in Equation (1) is only dependent on the haplotypes present and their frequencies through their contribution to *q_i_*.

To incorporate sequencing error, we modify the expected WSAF such that the allele frequency of ‘REF’ read as ‘ALT’ is $(1-q_{i})e$, and the allele frequency of ‘ALT’ read as ‘REF’ is *q_ie_*. Thus, the adjusted expected WSAF becomes:

(4) $$\pi_{i}=q_{i}+(1-q_{i})e-q_{i}e=q_{i}+(1-2q_{i})e.$$

We model over-dispersion in read counts relative to the Binomial using a Beta-binomial distribution. Specifically, the read counts of ‘ALT’ are identically and independently distributed (i.i.d.) Bernoulli random variables with probability of success *v_i_*; i.e. $a_{i}∼Binom(a_{i}+r_{i},v_{i})$, and $v_{i}∼Beta(\alpha ,\beta )$, where $E(v_{i})=\alpha /(\alpha +\beta )=\pi_{i}$. This is achieved by setting $\alpha =c\cdot \pi_{i}$ and $\beta =c\cdot (1-\pi_{i})$, such that the variance of the WSAF is inversely proportional to *c*. Combined, we have:

(5) $$L(q_{i}|e,D)\propto \frac{\Gamma (a_{i}+c\cdot \pi_{i})\Gamma (r_{i}+c\cdot (1-\pi_{i}))}{\Gamma (c\cdot \pi_{i})\Gamma (c\cdot (1-\pi_{i}))}.$$

#### *2.2.2* Prior distributions

Rather than model the number of strains, *n*, directly, we take the approach of fixing *n* to be at the upper end of what can realistically be inferred (typically 5), using a skewed prior for proportions (such that typically only 1–2 strains might be at appreciable frequency) and then discarding strains inferred to have a proportion less than some critical amount (e.g. 1 percent).

To achieve this, we model the proportions of the *n* strains through a log titer, *x_k_*, drawn from a $N(\eta ,\sigma^{2})$ prior. The proportion of strain *k*, *w_k_*, is given by

(6) $$w_{k}=\frac{\;\text{exp}\;(x_{k})}{\sum_{j=1}^{n}\;\text{exp}\;(x_{j})},$$

and the prior density is given by the distribution function for the value of **x**.

Haplotypes, **h**, are modelled as being generated independently from the reference panel by the Li and Stephens (2003) process, though with a rate of mis-copying that is independent of the panel size. That is, under the prior, a path through the reference panel is sampled as a Markov process where recombination enables switching between members of the reference panel and mis-copying allows the allelic state of the haplotype within the sample to differ from the allelic state of the reference panel haplotype being copied at the site. The transition probability of switching from copying reference haplotype *a* to reference haplotype *b* is $\left(1-\text{exp}\;(-G\psi_{i}))/|\Xi \right|$, where *ψ_i_* is the genetic distance (in Morgans) between sites *i* and *i* + 1, *G*, is a scaling factor (described below in more detail) and |Ξ| is the size of the reference panel. Note that unlike the original model, the recombination or switching rate is not dependent on sample size.

For miscopying, let *ξ_k_* denote the state of the sequence in the reference panel Ξ that *h_k_* is copying from at given site and *μ* denote the probability of miss-copying:

$$\{\begin{array}{cc} P(\xi_{k}=h_{k}) & =1-\mu ,\\ P(\xi_{k}\neq h_{k}) & =\mu . \end{array}$$

As above, this is a simple reparamterization of the original model, but where the miscopying rate is independent of the sample size. The emission probabilities are given by the convolution of the reference panel paths and the miscopying process, strain proportions and the read error rate.

### 2.3 Inference

To infer the haplotypes present in a mixed infection and their relative proportions we use a Markov chain Monte Carlo (MCMC) approach. We learn the relative abundance of each strain by exploiting signatures of within-sample allele frequency imbalance, using a Metropolis-Hastings algorithm, which samples proportions (w) given haplotypes (h). While updating w, we rely on ‘painting’ strain haplotypes with a reference panel to recover individual haplotype structure. Our Gibbs sampler updates h for a given w by adjusting either a single sequence or a pair of haplotypes (to speed up convergence). We iterate through these MCMC steps in a random order. Details can be found in the Supplementary Material.

### 2.4 Implementation details

- **Number of strains**. As described above, we aim to infer more strains than are actually present, starting the MCMC chain with a fixed *n*, which has a default of 5. At the point of reporting, we discard strains with a proportion less than a fixed threshold, typically 0.01.
- **Parameters**. The parameter *c* (Equation (5)) reflects how much data are available. The mean coverage of the validation dataset ranges from 106.20 to 147.04, with a mean of 124.487. In practice, we set the parameters *c* = 100; *η* = 0, $\sigma^{2}=5$ which are adjusted accordingly when working with extremely unbalanced samples (Section 2.2.2 and Supplementary Material). We set the read error rate as 0.01 and the rate of mis-copying as 0.01.
- **Recombination rate and scaling**. We assume a uniform recombination map, where the genetic distance between loci *i* and *i* + 1 is computed by $\psi_{i}=D_{i}/d_{m}$ where *D_i_* denotes the physical distance between loci *i* and *i* + 1 in nucleotides and *d_m_* denotes the average recombination rate in Morgans bp^–^^1^. We use the recombination rate for *P. falciparum* of 15 000 base pairs per centiMorgan as reported by Miles *et al.* (2016). The recombination rate is scaled by a factor *G*, which reflects the effective population size, rate of inbreeding and size and relatedness of the reference panel. In practice, we deconvolve over 1 million markers in field samples. We use a value of *G* = 20 to ensure small values for recombination probabilities between any two markers, with a mean of 0.015. A large value of *G* relaxes the reference panel constraint, becoming an LD free model when *G* is infinity. In practice, inference seems largely insensitive to choices around this parameter. The scaled genetic distance Gψ is used to compute the transition probability of switching from copying reference haplotype *a* to reference haplotype *b* (see Supplementary Material for details).
- **Update without linkage disequilibrium**. For initializing the chain, or if the markers present are very widely spaced, linkage disequilibrium can be ignored, which is equivalent to setting the genetic distance between adjacent loci to be infinitely high. Under these circumstances, the haplotype updates become much simpler and depend only on the population-level allele frequency (PLAF), for example as estimated from the reference panel or provided independently.
- **Reporting** We aim to provide users with a single point estimate of the haplotypes and their proportions, although the full chain is also available for analysis. To achieve this we report values at the last iteration—i.e. we report a single sample from the posterior. However, to measure robustness, we typically repeat the deconvolution with multiple random starting points. We use a majority vote rule on the inferred number of strains; we then select the chain with the lowest average deviance (after removing the burn-in) as our estimate. The deviance measures the difference in log likelihood between the fitted and saturated models, the latter being inferred by setting the WSAF to that of the observed values. These parameters can be modified by users to achieve a preferred balance between computational speed and confidence. By default, we set the MCMC sampling rate as 5, with the first 50% of samples removed as burn in and 800 samples used for estimation.
- **Reference panel construction**. To infer clonal samples for the reference panel we use the Pf3k project data, running the algorithm without LD on all samples and identifying those with a dominant haplotype (proportion > 0.99) as clonal. These clonal samples are grouped by region of sampling to form location-specific reference panels. In addition, we have included a number of reference strains, described in more detail below.

## 3 Validation and performance

**Fig. 1.**
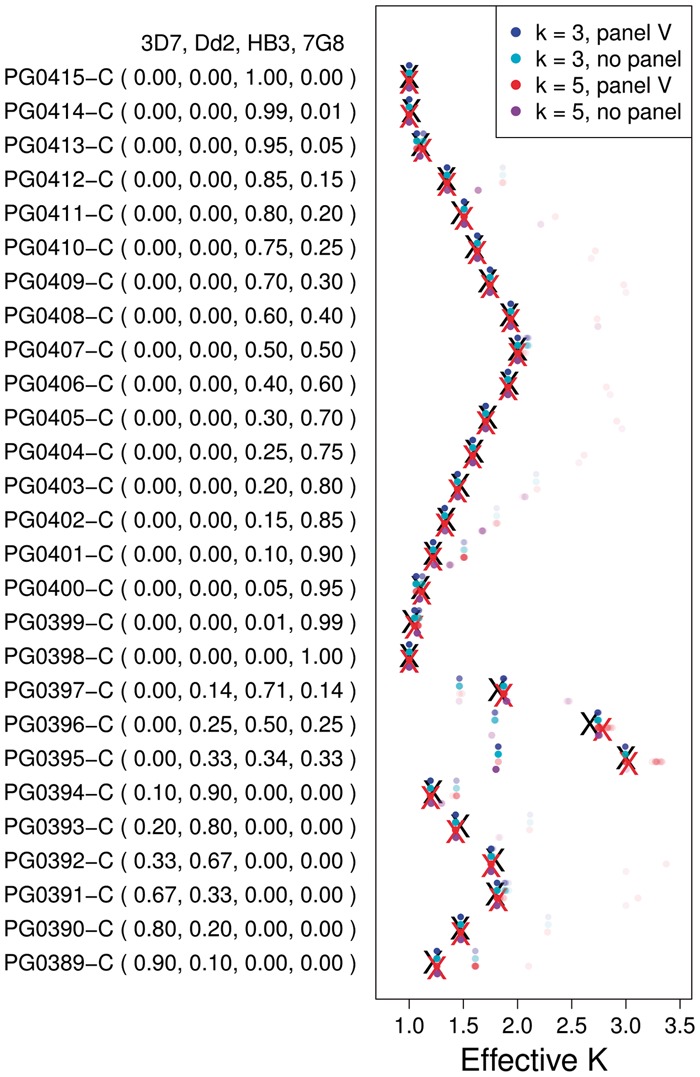
Experimental validation and effective number of strains inferred by DEploid. We use Reference Panel V to deconvolve the same lab-mixed samples, assuming 3 and 5 strains within a sample. Each experiment is repeated without a reference panel. Black crosses indicate the true effective number of strains. Red crosses indicate median values obtained from 30 replicates when using a panel and assuming that the maximum number of strains is 5. The coloured dots show the inferred effective number of strains across replicates with intensity proportional to fraction

As validation we used a set of *in vitro* mixtures created by Wendler (2015) to simulate mixed infections. DNA was extracted from four laboratory parasite lines: 3D7, Dd2, HB3 and 7G8, experimentally mixed in different proportions (see Fig. 1), and submitted to the MalariaGEN pipeline (MalariaGEN, 2008) for Illumina sequencing and genotyping (Manske *et al.*, 2012).

This dataset only contains two unmixed samples, which is insufficient for constructing a reference panel. Moreover, the *P.falciparum* genetic crosses project (Miles *et al.*, 2016) found that due to sequencing error, mapping error and variation among variant calling methods, genotype calls vary at the same locus for the same strain of *P.falciparum*. To create a baseline reference haplotype for each strain we therefore considered multiple samples that contains the same parasite strains.

*Inferring haplotypes for Dd2 strain.* Since 3D7 is the reference strain, we assume that strain Dd2 is the only source of ‘ALT’ reads in samples PG0389-C to PG0394-C. Assuming markers are independent from each other, let *y* be the read count for ‘ALT’ allele and *x* be the total read count weighted by the Dd2 mixing proportion (see Fig. 1 in brackets), we use a regression model ($y=\beta_{0}+\beta_{1}x$) to infer the Dd2 genotype: 1 if *β*_1_ is significant with *P*-values below 0.001; 0 otherwise.

*Inferring haplotypes for HB3 and 7G8.* Similarly, for samples PG0398-C to PG0415-C, we let variables *x*_1_, *x*_2_ be the coverages weighted by the mixing proportions of HB3 and 7G8 respectively; we use a regression model ($y=\beta_{0}+\beta_{1}x_{1}+\beta_{2}x_{2}$) to infer the genotypes of HB3 and 7G8: HB3 is 1 if *β*_2_ is significant with *P*-values below 0.001; 0 otherwise; similarly for 7G8.

To investigate how the haplotype inference accuracy is affected by the quality of the reference panel (in terms of having haplotypes close to those present in the samples) we experimented with deconvolving the 27 lab-mixed samples with the following reference panels:

- Panel I: five Asian and five African clonal strains from the Pf3k resource: PD0498-C, PD0500-C, PD0660-C, PH0047-Cx, PH0064-C, PT0002-CW, PT0007-CW, PT0008-CW, PT0014-CW, PT0018-CW;
- Panel II: panel I with the addition of HB3;
- Panel III: panel II with the addition of 7G8;
- Panel IV: panel III with the addition of Dd2;
- Panel V: 3D7, HB3, 7G8 and Dd2 strains (the perfect reference panel for the lab mixtures);
- Panel VI: Panel I with the addition of six (three each) clonal strains from Asia and Africa: PH0193-C, PH0283-C, PH0305, PT0060-C, PT0146-C and PT0158-C (a typical reference panel for field samples of unknown geographical origin).

### 3.1 Accuracy

Our validation experiments use variant calls of these 27 lab-mixed *in vitro* samples, which are produced by the Pf3k pipeline based on GATK best practices (McKenna *et al.*, 2010) on 2512 field isolates and 128 lab samples. The filter threshold is set at a level for which false positive genotype calls (calling a variant that doesn’t exist) and false negative calls (not calling a true variant) are equal. From the 18 570 high-quality biallelic SNPs, we observe a small number of heterozygous sites with high coverage, which can potentially cause our model to over-fit the data with additional strains. After the filtering step (see Supplementary Material for details), we deconvolve the remaining 17 530 sites for all experiments in the rest of this section, unless specified otherwise.

#### *3.1.1* Proportions and number of strains

**Fig. 2.**
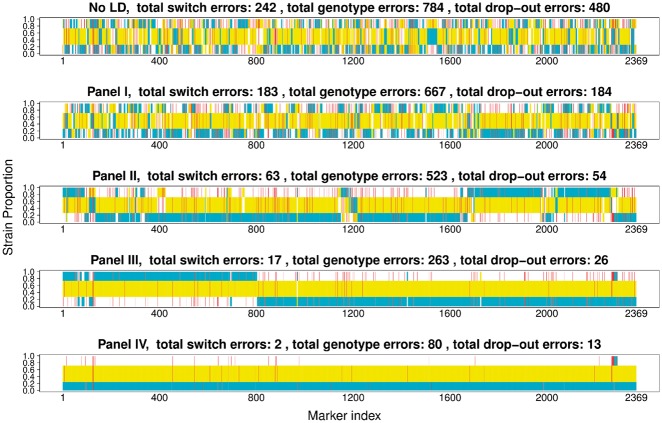
Comparison of true and inferred haplotypes for Chromosome 14 (2369 SNPs) in sample PG0396-C without linkage disequilibrium (top) and using Reference Panels I to IV (from the second to the bottom). Reference Panel V gives results equivalent Panel IV and Panel VI gives results similar to Panel I. Red bars mark wrongly inferred positions. The yellow, cyan and white background label the haplotype segments from strains 7G8, HB3 and Dd2 respectively. The switch errors are obtained by counting the changes of a strain segment mapped to reference strains; the genotype errors are the discordance between the strain and the mapped reference segments. These results demonstrate the value of including reference strains similar to those present in the sample being analyzed

To evaluate accuracy of estimates we used the effective number of strains, calculated as $1/\sum w_{i}^{2}$, which reflects the number and proportions of strains present. We also assessed sensitivity of estimates to the number of fitted strains (3 or 5). Typically, we find consistent inference of the effective number of strains regardless of the assumption of number of strains or the use of LD information (see Fig. 1). The deviance between the expected and inferred proportions per sample is bounded by the inverse of the deviation between expected and observed effective number of strains (derived in the Supplementary Material), with an average of 0.023. We also explored the quality of estimates for deconvolving a mixture of 3 strains (Dd2/7G8/HB3) from different reference panels. In all cases we estimated the number and proportion of strains accurately, for example Figure 2 shows the proportions of strains Dd2/7G8/HB3 as being accurately inferred as approximately $\frac{1}{4},\;\frac{1}{2}$ and $\frac{1}{4}$.

#### *3.1.2* Haplotypes

**Fig. 3.**
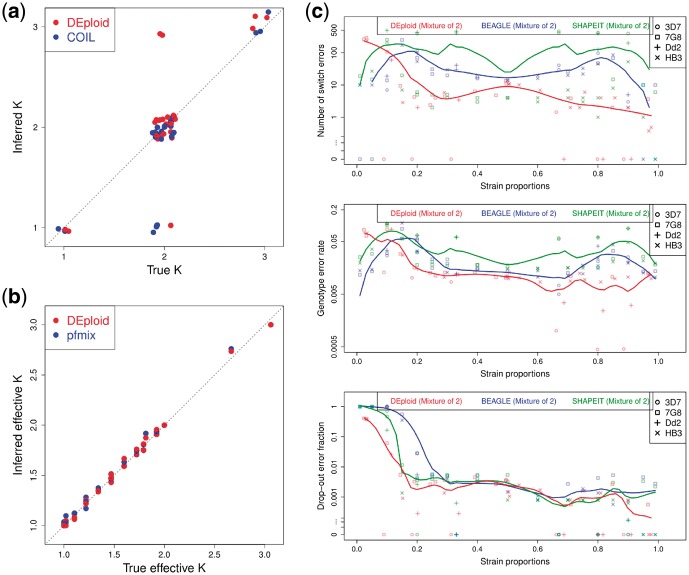
Comparison of DEploid and existing tools (COIL, pfmix, BEAGLE and SHAPEIT). (**a**) Estimates for the number of strains present in each mixed infection (artificially mixed in the lab) as given by COIL and DEploid. (**b**) Comparison of the inferred effective number of strains of each mixture as given by pfmix and DEploid. (**c**) Relationship between strain proportions and haplotype inference accuracy in the experimental validation for DEploid and BEAGLE/SHAPEIT (only mixtures of two strains). We used Reference Panel V to deconvolute all 27 samples with default settings. Each point represents a deconvolved haplotype with 17 530 sites. Point shape refers to strain and colour indicates the method applied. We use LOESS smoothing to show the trend of error versus strain proportion. Top panel shows switch error rate, the middle panel indicates genotyping error rate and the bottom panel indicates genotyping error rate through strain dropout. Note that zero switch error is represented as points below one. In summary, we find that DEploid results for the number of strains and relative proportions in a mixture are comparable to those achieved by existing methods, while inferred haplotypes are considerably better than from other methods

To understand how the inferred haplotypes relate to the correct haplotypes we use a dynamic programming algorithm to identify switch errors, genotype discrepancies and regions where one or more haplotype has been dropped out (a distinct form of genotype error). Example deconvolutions are shown in Figure 2 and an overview of all experiments is shown in Figure 3. From our assessment of haplotype inference, we conclude:

- The inference of relative proportions does not seem to be affected by the use of linkage disequilibrium information from the reference panel or its closeness to the samples being analyzed (Fig. 2).
- The accuracy of haplotype inference is, however, dependent on having an appropriate reference panel in terms of relatedness to the samples being analyzed (Fig. 2).
- The strain proportion affects haplotype inference (see Fig. 3). Our method infers strains with proportions over approximately 20% with high accuracy, but struggles with minor strains due to insufficient data, in particular at sites when the minor strain carries the alternative allele and the dominant strain carries the reference allele (see Fig. 3).

### 3.2 Comparison to existing methods

A mixed infection can be completely described by the number of co-existing strains, their relative proportions and their associated haplotypes. Existing methods for characterizing mixed infections are limited to providing a summary statistic of relative inbreeding ($F_{\text{ws}}$, Manske *et al.*, 2012), inferring the number of strains (COIL), or simultaneously inferring the number of strains and their proportions (pfmix, O’Brien *et al.*, 2016). DEploid is the only method that can also estimate haplotypes although it can be argued that conventional tools for phasing diploid organisms (BEAGLE, SHAPEIT) could be used to deconvolute mixtures of two strains.

In this section, we use the same dataset (27 samples) to compare DEploid with all the methods mentioned above (see Supplementary Material for details). Our method correctly infers the number of strains in 24 out of 27 samples when a reference panel is provided. In comparison, COIL correctly infers the number of strains in 23 samples. We notice that both methods struggle to identify strains whose relative proportions is below 5% (Fig. 3a). Specifically, both methods fail to detect the minor strain at 1% in sample PG0414-C. However, while COIL typically fails to identify strains with a proportion below 5%, DEploid tends to over-fit the minor strain with an additional component.

The method pfmix infers the number of strains and proportions solely from the allele frequency imbalance within sample: It infers the strain proportions assuming different number of strains (from one to eight), then uses the Bayesian information criterion to choose the best model. As we were unsuccessful in our attempt to use pfmix with our dataset, we ignore the model selection step of pfmix, and infer proportions directly with fixed number of strains. Similar to the comparison shown in Figure 1, we compute the observed and expected effective number of strains of each sample, and find consistent results between DEploid and pfmix.

We also investigated the use of BEAGLE and SHAPEIT for deconvolving haplotypes in mixtures of two strains. BEAGLE and SHAPEIT would implicitly assume a 50:50 distribution of alleles, since they have been designed for diploid organisms. Both methods worked well for balanced mixtures (i.e. with proportions between 40 and 60%) as they mimic a diploid sample. However, as strain proportions became more unbalanced, accuracy degraded and both methods incorrectly inferred heterozygous sites as homozygous, introducing a bias towards inferring the haplotypes of dominant strains. We observed that strains with a relative proportion below 20% were always masked out by the dominant strain (Fig. 3c).

### 3.3 Simulation from field samples

**Fig. 4.**
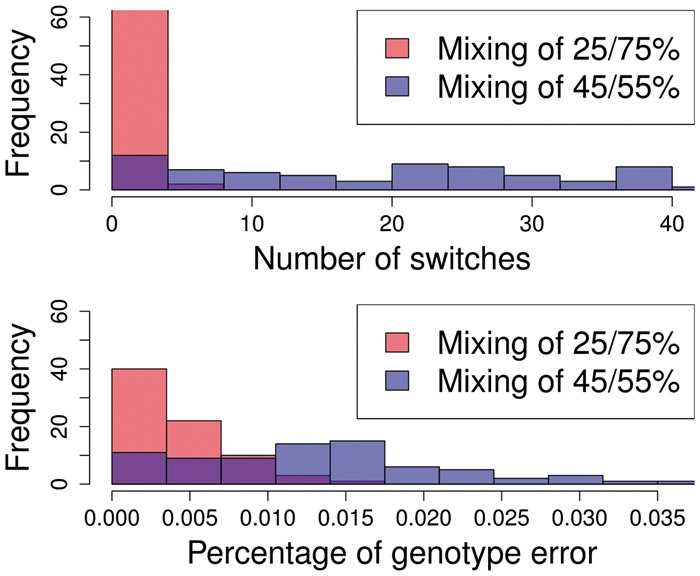
Histograms of switch error and genotype error across 78 simulated Pf3k samples. We excluded four cases out of the 100 experiments where simulated haplotypes were over 99% identical and 18 cases where average coverage was below 20

The lab strain mixture reflects an artificial situation that is unlikely in the field. To assess the performance of DEploid in realistic settings, we created *in silico* mixtures from 212 clonal samples of Asian origin with two proportions (25/75% and 45/55%) for 8071 sites from Chromosome 14. A further 20 randomly chosen samples were used for the reference panel. To simulate data, we used empirical read depths and drew read counts for the two alleles from the binomial proportions given by Equation (4). DEploid correctly recovered the number of strains and proportions. As expected, we observed more switches and genotype errors in 45/55% mixtures than 25/75% mixtures, with means of 24.3 and 0.57 for switch errors, and 0.013 and 0.0042 for per-site genotype errors (combining isolated and strain drop-out errors) respectively (Fig. 4). In summary, because field samples are likely to contain haplotypes much more closely related to each other than the artificial lab strain mixtures, we consequently expect high genotype accuracy and, at least for samples with unbalanced mixtures, very low switch error rates.

### 3.4 Run-time

The complexity of our program is $O(lm^{2})$, where *m* and *l* are the number of reference strains and sites respectively. In practice, we recommend dividing samples into distinct geographical regions to perform deconvolution. We then compute the number of differences between clonal strains, and use the 10 most different local clonal strains as reference panel. The run time for deconvolution a field sample range between 1 and 6 h, depending on the number variants in a sample: For example, it takes $5\frac{1}{2}$ h to process sample QG0182-C over 372 884 sites. We give worked examples of deconvolving mixed infections from *in vitro* samples in the Supplementary Material.

## 4 Discussion

The program DEploid and its analysis pipeline has been originally developed for *P.falciparum* studies. Nonetheless, with some parameter changes, DEploid can potentially be used for deconvolution of other datasets with a mixture of samples from a single species, for example on data from *Plasmodium vivax* (Pearson *et al.*, 2016) or bacterial and viral pathogens. However, it is likely that fine-tuning of parameters (for example, relating to mis-copying rates, read over-dispersion and recombination), filtering of datasets to remove poorly genotyped variants and collation of appropriate reference panels will be necessary to achieve effective results. We show examples and discuss the effect of fine-tuning parameters in the Supplementary Material.

There are several limitations of the current implementation, the greatest of which is the quadratic scaling with reference panel size. Note that a typical reference panel from field samples will not guarantee all haplotype structure representative of the population is present. Therefore, it would be ideal to include as many reference strains as possible. However, this approach is computationally prohibitive. In practice, current approaches to related problems such as haplotype phasing (Delaneau *et al.*, 2012) or inference from low-coverage sequencing experiments (Davies *et al.*, 2016) typically aim to select a few candidate haplotypes (which might be a mosaic) from a reference panel. Alternatively, the reference panel data can itself be approximated, for example through graphical structures, as in BEAGLE (Browning and Browning, 2007), or represented through structures that enable efficient computation (Lunter, 2016). Our current implementation also only considers biallelic variants. To reconstruct the complete haplotype, we should also consider structural variants such as insertions and deletions, which will require tailored error models. Such extensions will be pursued in future work.

Recently, single molecule sequencing with long-read data has become available, for example through PacBio or Oxford Nanopore Technologies. Read lengths of several kilobases can provide information on linkage between variants within a sample, which is typically very limited from short reads, hence not considered here. Similarly, single-cell technologies can provide long-range, though typically very patchy information. Although integration of such data types will require modifications to algorithms and technology-specific error models, they also provide great potential to provide highly accurate and genome-wide characterization of multiple strains within samples.

## Supplementary Material

Supplementary Data
