# Supplementary material for: Deconvolution of multiple infections in Plasmodium falciparum from high throughput sequencing data
Source: Bioinformatics. 2017 Aug 22;34(1):9–15. doi: 10.1093/bioinformatics/btx530 (PMC5870807; doi:10.1093/bioinformatics/btx530)
Supplement: Supplementary Data [file bioinfosupplement_btx530.pdf]

Deconvolution of mixed infections in *Plasmodium falciparum* from high throughput sequencing data: Supplementary Material

Sha Joe Zhu, Jacob Almagro-Garcia and Gil McVean

Contents

S1 Technical details **S1–1**

S1.1 Inference . . . S1–1

S1.1.1 Metropolis-Hastings update for proportions . . . S1–1

S1.1.2 Gibbs update for single haplotype . . . S1–1

S1.1.3 Gibbs update for a pair of haplotypes . . . S1–2

S1.2 Proof of the bound of the deviance . . . S1–3

S2 DEploid examples **S2–1**

S2.1 Data exploration and filtering . . . S2–1

S2.2 Deconvolution of sample PG0396-C . . . S2–2

S2.3 Deconvolution of extremely unbalanced samples . . . S2–7

S3 Assessing coverage requirements **S3–1**

S4 Comparison to existing methods **S4–1**

S4.1 `COIL` . . . S4–1

S4.2 `pfmix` . . . S4–1

S4.3 `BEAGLE` . . . S4–1

S4.4 `SHAPEIT` . . . S4–2

S1 Technical details

S1.1 Inference

S1.1.1 Metropolis-Hastings update for proportions

We update $\mathbf{w}|n$, through the underlying log titres, $\mathbf{x}|n$. Specifically, we choose i uniformly from n and propose new x_i's from $x_i' = x_i + \delta x$, where $\delta x \sim N(0, \sigma^2/s)$, and s is a scaling factor. The new proposed proportion is therefore $\frac{\exp(x_k')}{\sum_{k=1}^{n} \exp(x_k')}$. Since the proposal distribution is symmetrical, the Hastings ratio is 1. A new update is accepted with probability

$$\min\left(1, \frac{P(\mathbf{w}'|n)}{P(\mathbf{w}|n)} \frac{L(\mathbf{w}', \mathbf{h}|\Xi, e, D)}{L(\mathbf{w}, \mathbf{h}|\Xi, e, D)}\right).$$

S1.1.2 Gibbs update for single haplotype

We choose haplotype strain s uniformly at random from n strains to update. At each site, given the current proportions, we can calculate the likelihood of the 0 and 1 states. To achieve this, we first remove it from the current WSAF, i.e. subtract $w_s \cdot h_s$ from Eqn. (3), which gives

$$q_{i,-s} = \sum_{k \neq s} w_k \cdot h_k = \text{Eqn. (3)} - w_s \cdot h_s. \tag{S1.1}$$

Therefore, updating the allelic state of strain s to 0 and 1, the expected WSAF becomes

$$q_{i,h_s=0} = \text{Eqn. (S1.1)} \tag{S1.2}$$

$$q_{i,h_s=1} = \text{Eqn. (S1.1)} + w_s \times 1. \tag{S1.3}$$

We substitute Equations (S1.2) and (S1.3) into Eqn. (5) after adjustment for read error.

Given the structure of the hidden Markov model and the above likelihoods, the forward algorithm can be used to sample a path through the reference panel, and subsequent mis-copying, efficiently from the marginal posterior distribution. In effect, the reference panel is used as a prior on haplotypes present in the sample (with recombination creating a mosaic of the different haplotypes) and the mis-copying process allows for recent mutation, recurrent mutation, gene conversion and some types of technical error. Figure S1.1 illustrates the approach.

Our goal is to sample the allelic states for the haplotype to be updated from the posterior distribution given the reference panel, the prior on paths through this panel, the rate of mis-copying, and the error distribution for read counts at sites. This can be achieved through the use of the forward algorithm within

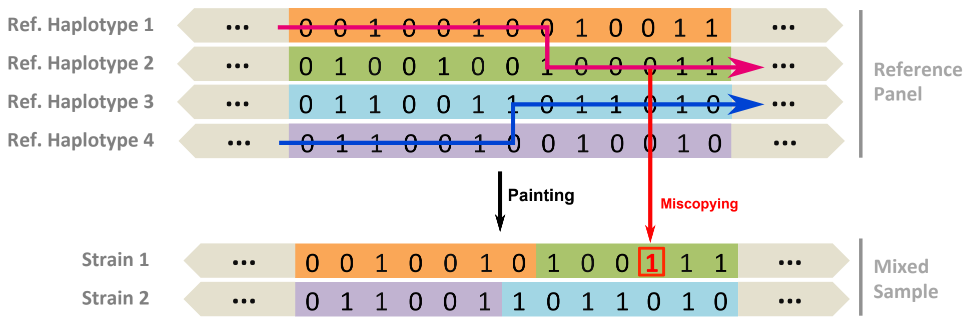

Figure S1.1: The Li and Stephens (2003) algorithm as applied to the problem of multiple strain inference. Strain 1 haplotype is made up from reference haplotype segments of 1 and 2; and strain 2 haplotype is made up from reference haplotype segments of 3 and 4. With mis-copying, we allow strain states differ from the path: At the third last position of strain 1, the path is copied from reference haplotype 2, with the state of 0.

the hidden Markov model formulation.

Let $F(j,i)$ be the value in the forward matrix for reference strain j at site i. The emission probabilities are calculated as:

$$L(\xi_{j,i}) = \begin{cases} (1-\mu)L(q_{i,h_{s=0}}) + \mu L(q_{i,h_{s=1}}) & \text{if } \xi_{j,i} = 0 \\ \mu L(q_{i,h_{s=0}}) + (1-\mu)L(q_{i,h_{s=1}}) & \text{if } \xi_{j,i} = 1. \end{cases} \tag{S1.4}$$

We initialise the forward matrix with

$$F(j,1) = \frac{1}{|\Xi|} L(\xi_{j,1}). \tag{S1.5}$$

We use ψ_i to denote the genetic distance, G, the scaling factor and ρ'_i to denote the probability of **no** recombination between site i and $i+1$, we have $\rho'_i = \exp(-G\psi_i)$. The elements of the forward matrix can then be computed from:

$$F(j,i+1) = \big(\rho'_i F(j,i) + \frac{1-\rho'_i}{|\Xi|} \sum_{k=1}^{|\Xi|} F(k,i)\big) L(\xi_{j,i+1}) \tag{S1.6}$$

Once the forward matrix has been filled, we then sample first a path through the matrix using the standard approach and, conditional on this path, the mis-copying process. The resultant haplotype is therefore drawn, as desired, from the posterior distribution of haplotypes.

S1.1.3 Gibbs update for a pair of haplotypes

In order to improve mixing, we also perform Gibbs-sampling updates for pairs of haplotypes (given current proportions). The algorithm proceeds as for the single-haplotype update, though with a larger state space. First, we sample a pair of haplotypes, s_1 and s_2, uniformly. As in Equation (S1.1), we first remove their states from the WSAF:

$$\begin{aligned} q_{i,-s_1,-s_2} &= \sum_{k \neq s_1, s_2} w_k \cdot h_k \\ &= \text{Eqn. (3)} - w_{s_1} \cdot h_{s_1} - w_{s_2} \cdot h_{s_2}. \end{aligned} \tag{S1.7}$$

Considering all four possible combination of genotypes, we can then write down the expected WSAF:

$$q_{i,h_{s_1}=0,h_{s_2}=0} = \text{Eqn. (S1.7)} \tag{S1.8}$$

$$q_{i,h_{s_1}=0,h_{s_2}=0} = \text{Eqn. (S1.7)} + w_{s_1} \times 1 \tag{S1.9}$$

$$q_{i,h_{s_1}=0,h_{s_2}=1} = \text{Eqn. (S1.7)} + w_{s_2} \times 1 \tag{S1.10}$$

$$q_{i,h_{s_1}=0,h_{s_2}=1} = \text{Eqn. (S1.7)} + w_{s_1} \times 1 + w_{s_2} \times 1. \tag{S1.11}$$

Substituting expressions. (S1.8) to (S1.11), into Eqn.(5), we then obtain their associated likelihoods.

As in the single-haplotype update, the hidden Markov model formulation enables us to sample a pair of paths through the reference panel (and the mis-copying process) efficiently from the marginal posterior distribution using the forward algorithm, that is given the other haplotypes and their inferred proportions.

Similar to the previous section, we consider updating the allelic states of the pair of haplotypes s_1 and s_2 given the reference panel, the prior on paths through this panel, the rate of mis-copying, and the error distribution for read counts at sites. However, here the underlying state is the pair of paths through the reference panel (hence the quadratic nature of the algorithm).

At each site, the emission probabilities for each pair of paths (where the two paths may be copying the same haplotype in the reference panel) are given by combining the expected WSAF calculation of Equations 11-14 in the main text with the mis-copying process, as for the single haplotype case. For the sake of brevity we omit the equations as they follow naturally, noting that the two haplotypes are not exchangeable due to their having different proportions. As above, we complete the forward matrix, sample a pair of paths through the matrix and then the mis-copying process to provide a sample of strain haplotypes from the marginal posterior.

S1.2 Proof of the bound of the deviance

Let k denote the strain index, w_k and $\hat{w}_k$ be the expected and inferred proportion of strain k receptively. Let the expression $\sum_k w_k^2$ to denote the inverse of the effective number of strains. We find the deviance between the expected and inferred proportions are bounded by the following inequality:

$$\sum_k |(w_k - \hat{w}_k)^2| \leq |\sum_k w_k^2 - \sum_k \hat{w}_k^2|, \tag{S1.12}$$

where

$$LHS = \sum_k \sqrt{((w_k - \hat{w}_k)^2)^2},$$

and

$$RHS = |\sum_k w_k^2 - \hat{w}_k^2| = \sum_k |w_k^2 - \hat{w}_k^2| = \sum_k \sqrt{(w_k^2 - \hat{w}_k^2)^2}.$$

By deriving $((w_k - \hat{w}_k)^2)^2 \leq (w_k^2 - \hat{w}_k^2)^2$, we show that the inequality (S1.12) holds.

S2 DEploid examples

Our program DEploid is freely available, including C++ source code at https://github.com/mcveanlab/DEploid under the conditions of the GPLv3 license. A detailed document can be found at http://deploid.readthedocs.io/en/latest/. An R version is available at https://github.com/mcveanlab/DEploid-r.

S2.1 Data exploration and filtering

As a demonstration, we show some examples of mixed *P. falciparum* genome deconvolution using one of the reference panels described in the main article (Reference Panel V). All data used in this chapter is avaiable at https://github.com/shajoezhu/DEploid-Supplementary-Materials. First, we use an R script to plot and explore the data of a mixed sample. For instance, sample PG0395-C is a mixture of strains HB3, 7G8 and Dd2 with equal proportions, we run the following command

```
~/DEploid/utilities/dataExplore.r -vcf PG0395-C.eg.vcf.gz \
   -plaf labStrains.eg.PLAF.txt \
   -o PG0395-C
```

where "-vcf PG0395-C.vcf.gz" defines the input VCF file. Notice that, in this example, we assume all variant sites are single nucleotide polymorphisms, and every site is tagged with PASS at the QUAL column. The read counts of the reference and alternative alleles must be presented in the AD field at all sites; "-plaf labStrains.eg.PLAF.txt" contains the population allele frequencies calculated from total read counts (see main article Secion 2.1 Notations). This command produces two files with prefix specified by the flag "-o": 1. An image file of panel figures used for exploring the allele counts. 2. A text file listed potential outliers.

We find markers with high frequencies in both reference and alternative allele count can mislead our model to fit extra strains. We then use a threshold of $\geq 99.5\%$ coverage (default) to identify markers with extremely high allele counts. These markers often appears in clusters as a result of poor mapping. We further expand this list of potential "bad markers" by considering their nearest 10 neighbours on both sides along the genome, and identify the nearest markers as "to be excluded" if overlaps are found (see Figure S2.1).

The number of clusters in Figure S2.1 can provide some intuition on the number of mixed strains, see Figure S2.1 (a). However, this can be misleading, as the number of clusters does not always reflect the true number of mixed strains. In the example shown in Figure S2.1, the alternative vs reference counts figure only show two clusters, but the true number of mixture is three.

Similar to the alternative vs reference counts figure, the number of WSAF modes can be used as an indication of the number of strains and proportions within a field isolate (Figure S2.1 (a)). Again, this is not always true, Figure S2.1 shows that two modes of WSAF at approximately 0.33 and 0.66, but the true number of mixed strains is three, and the proportions are 1/3, 1/3 and 1/3. Consequently, a complex mixture of strains with even proportions is difficult to deconvolve without using a reference panel.

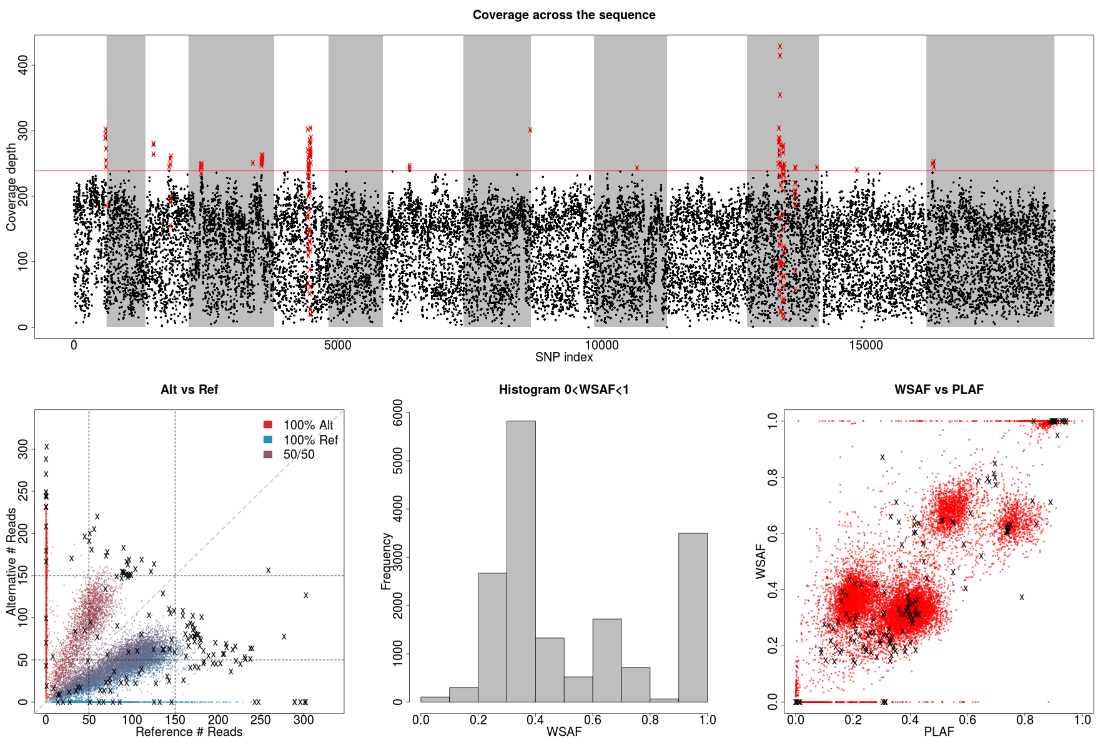

Figure S2.1: Data exploration of sample PG0395-C. From top to bottom, then from left to right, it shows: 1. Total allele counts across all markers. We use the threshold (red line) to identify markers with extremely high allele counts. Red crosses indicate markers that are filtered out, coloured in black in sub-figures 2 and 4. 2. Alternative read counts vs reference read counts. 3. Histogram of the allele frequencies within sample. Note that we exclude markers with WSAF strictly equal to 0s and 1s in the histogram. 4. Allele frequencies at the population level (PLAF) vs allele frequencies within the sample (WSAF).

S2.2 Deconvolution of sample PG0396-C

The following example shows a specific DEploid command to deconvolute the mixed sample PG0396-C:

```
dEploid -vcf PG0396-C.eg.vcf.gz \
    -plaf labStrains.eg.PLAF.txt \
    -exclude exclude.txt \
    -panel labStrains.eg.panel.txt \
    -seed 5 \
    -nSample 250 \
    -rate 8 \
    -burn 0.67 \
    -k 5 \
    -o PG0396-C_seed5k5
```

where flags “-vcf”, “-plaf” and “-exclude” are used in the same manner as in the previous example; “-panel labStrainsPanelFinal.txt” specifies a text file including haplotypes of lab strains 3D7, HB3, 7G8 and Dd2; options “-nSample”, “-rate” and “-burn” specify the total number of MCMC samples to take,

the sampling rate and the burn-in fraction of the MCMC chain respectively. For detailed documentation, please see http://deploid.readthedocs.io/en/latest/input.html.

We use DEploid to compute the posterior probabilities of the deconvolved haplotypes using the Li and Stephen's algorithm, and a utility R script to plot and interpret the output produced by DEploid. The following command is used to generate Figures S2.2 (a) – (e).

```
initialProp=$( cat PG0396-C_seed5k5.prop | tail -1 | sed -e "s/\t/ /g" )

dEploid -vcf PG0396-C.eg.vcf.gz \
   -plaf labStrains.eg.PLAF.txt \
   -exclude exclude.txt \
   -panel labStrains.eg.panel.txt \
   -painting PG0396-C_seed5k5.hap \
   -o PG0396-C_seed5k5 \
   -initialP ${initialProp}

~/DEploid/utilities/interpretDEploid.r -vcf PG0396-C.eg.vcf.gz \
   -plaf labStrains.eg.PLAF.txt \
   -exclude exclude.txt \
   -dEprefix PG0396-C_seed5k5 \
   -o PG0396-C_seed5k5 -ring
```

In this example, the second inferred haplotype (Figure S2.2 (d)) represents the 7G8 strain, which has relative proportion of 1/2. The remaining two strains have approximately equal proportions, which increase the difficulty to deconvolve. In practice, we find more switching errors when deconvolving samples containing strains at similar proportions, for example, Figure S2.2 (c) and (e) both show high probabilities of copying from strains HB3 and Dd2. More specifically, Figure S2.2 (c) panel 1 shows almost every position of inferred haplotype chromosome 1 is copying from strain HB3; but chromosome 2 is copying from strain Dd2; chromosome 3 is partly copying from strains HB3 and Dd2, with presence of one switching error. These observations suggest that our program DEploid can characterize the main genome diversities within a mixed samples, yet there is still room to improve to overcome the switching errors.

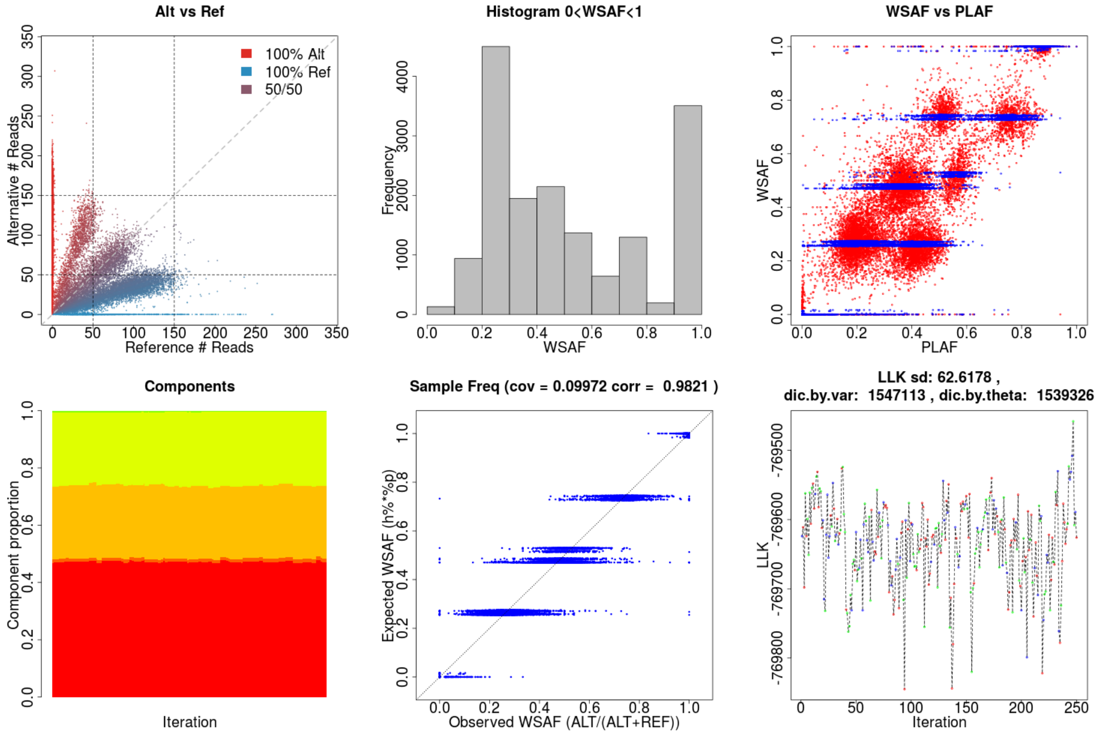

(a)

Figure S2.2: Sample PG0396-C deconvolution with Reference Panel V. (a) Diagnostic panels from the `DEploid` output. The top three panels recap the data exploration process, with an enhanced PLAF vs WASF plot: red dots show observed WSAF, which is calculated by read counts; blue dots show the expected WSAF inferred from our model (see Equation (3) in main article). The next three plots from left to right show: 1. MCMC samples for the strain proportions, with the fraction of each color indicating the proportion of a different strain at each MCMC sample. The three colored blocks suggest that there are three strains within sample PG0396-C, with proportions approximately 1/4, 1/4 and 1/2. 2. Expected WSAF vs observed WSAF. We use the correlation between the observed and expected WSAF as a sanity check for our model. A low correlation suggests poor fitting. 3. Log likelihood of the MCMC chain. This figure is used to indicate whether the MCMC has converged. The colored dots mark the likelihoods of the model when specific MCMC steps are used: updating the strain porportions, painting a single haplotype and painting a pair of haplotypes are marked in green red and blue respectively.

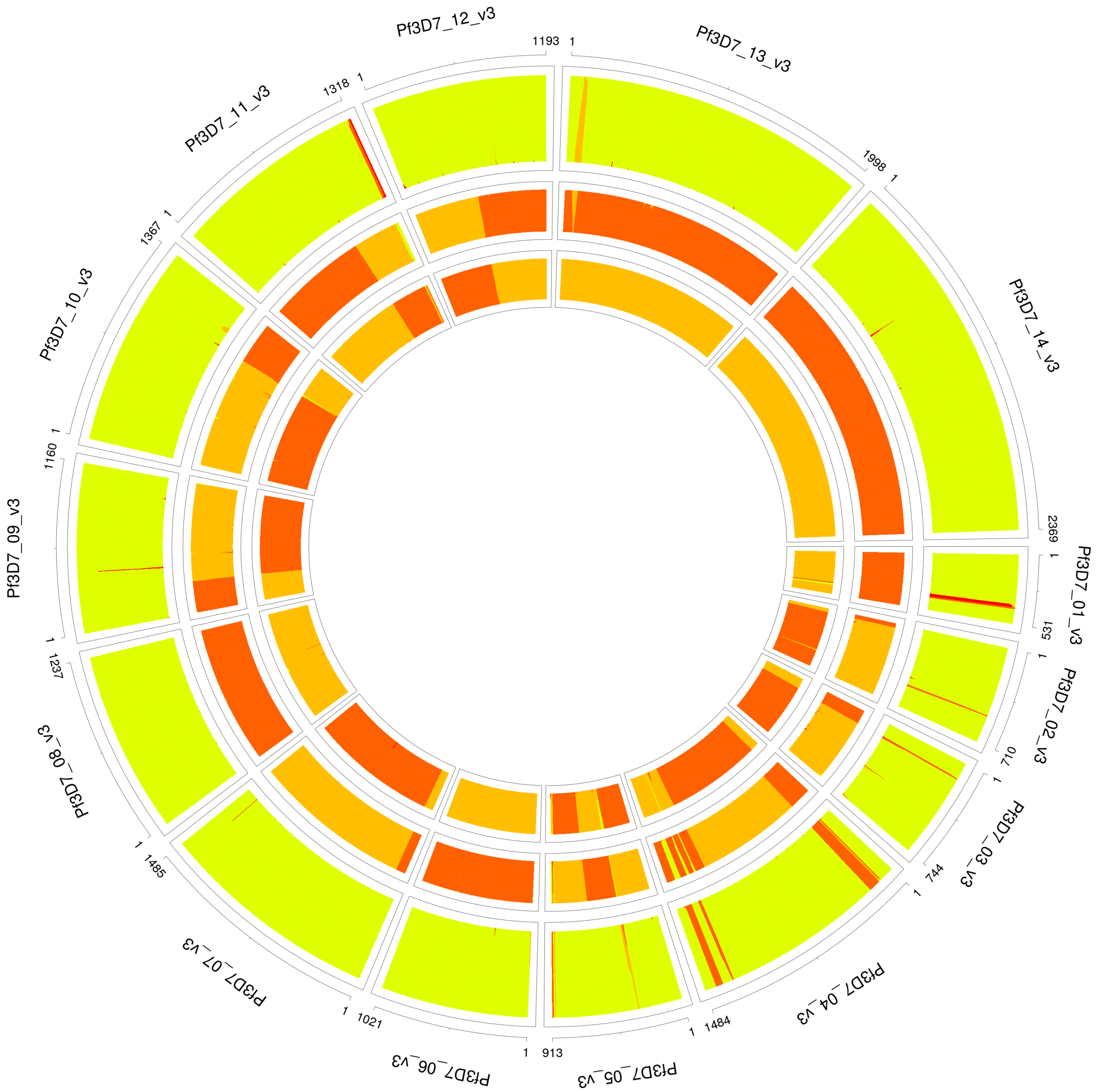

(b)

Figure S2.2: (b) Posterior painting probabilities for the deconvoluted strains when using the Reference Panel V. In each track, 3D7, HB3, 7G8 and Dd2 are represented by colors red, light orange, yellow and dark orange respectively. Each panel represents the posterior probability of a chromosome. Chromosomes 1 – 14 are ordered from 3pm clockwise. The width of each track is proportional to the inferred proportions in decreasing order towards the centre.

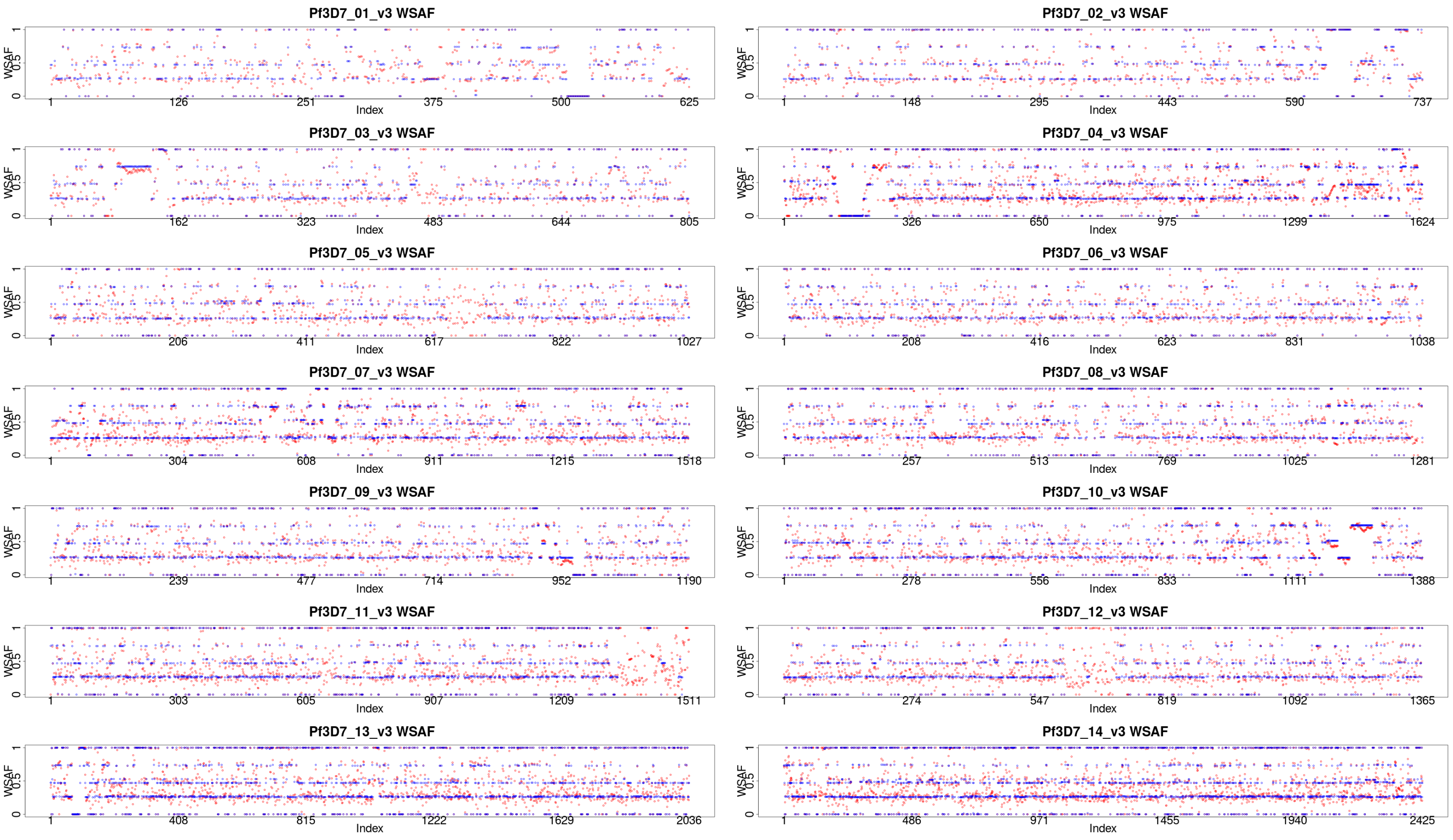

(c)

Figure S2.2: (c) Expected WSAF (blue) and observed WSAF (red) across the genome. This figure highlights the genome diversity within the mixed sample across the genome.

S2.3 Deconvolution of extremely unbalanced samples

While deconvolving the 27 lab-mixed samples with default settings, we overestimate the number of strains as three for samples PG0399-C, PG0400-C and PG0413-C 14, 19 and 18 times respectively from 30 replicates. In all cases, DEploid returns decent haplotype for the dominant strain, but chop the minor strain into two parts overall (in multiple segments), and fulfil them with haplotype segments from the dominant strain. This over-fitting procedure can achieve a higher likelihood than the model that we expect to reflect the truth. In order to improve the inference, we rerun the deconvolution 30 times with $\sigma^2 = 10$ and other parameters unchanged. Such modification uses a unimodal model (intuition is shown in Figure S2.3) to update the proportion, and ensures that a robust estimates for the dominate strain, which explains the main feature for extremely unbalanced mixtures. We estimate the number of strains correctly 26, 26 25 times out of 30 for samples PG0399-C, PG0400-C and PG0413-C respectively.

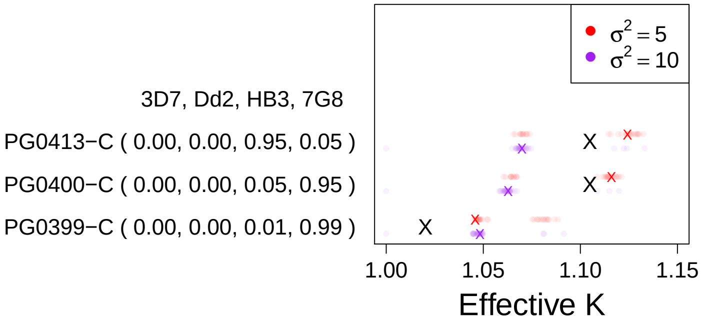

(a)

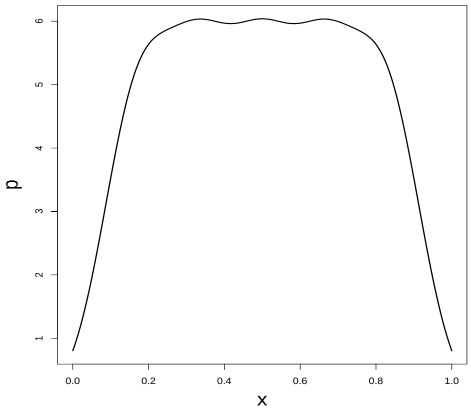

(b)

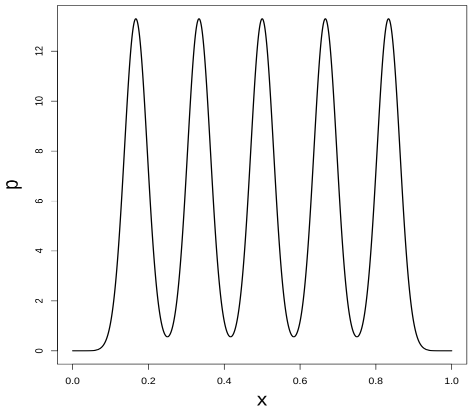

(c)

Figure S2.3: Figure (a) shows the changes in inferring the effective number of strains when using $\sigma^2 = 10$. The convolution of normal distribution $N(i/6, \sigma^2)$, for $i \in (1, 5)$, where $\sigma^2 = 0.09$ in (b) and $\sigma^2 = 0.03$ in (c). With a larger value of σ^2, the convolution behaves in a fashion of a unimodal distribution. Whereas a small value of σ^2 will result in a multi-modal fashion, which is more suitable for sparse updates.

S3 Assessing coverage requirements

In order to investigate how sensitive our method is to the sequence coverage, we simulate alternative and reference alleles read counts, and assess how the deconvoluted haplotypes compare to the truth. The previous section has shown that switching errors are common when two strain have similar proportions. Therefore here we consider to simulate data with uneven proportions.

We simulate total coverage from a Poisson distribution. Specifically we set the distribution mean to 10, 30, 40, and 50. Given the simulated total coverage, we then use a binomial distribution to simulate alternative allele counts using the expected WSAF calculated using Equation (3), where the allele states are of HB3 and 7G8, and the relative proportion used are 85% and 15% respectively, to mock sequence data of sample PG0402-C at different depths. Note that the expected WSAFs are adjusted using a constant error rate 0.01 (see Equation (4)). In this experiment, we only simulated data for chromosome 14, in particular at sites the PLAFs are non-zero (2425 sites in total). We then use DEploid to deconvolute the data, with a fixed number of strains of two.

We compare the simulated genotypes against the true genotypes of HB3/7G8: `0/0`, `0/1`, `1/0` and `1/1`. We first count occurrences of each true genotype. For each case, we then compare the inferred genotype against the truth, and count the number of times it was wrongly inferred, which is then divided by the true genotype occurrence to obtain the error rate. Figure S3.1 (a) – (c) shows high error rates for rare events (low coverage frequencies), and more importantly, the error rate decays when the mean coverage increases. When the coverage is above 50, we find low error rate in all cases, which suggests that expected coverage of the minor strain needs to be ≥ 7.

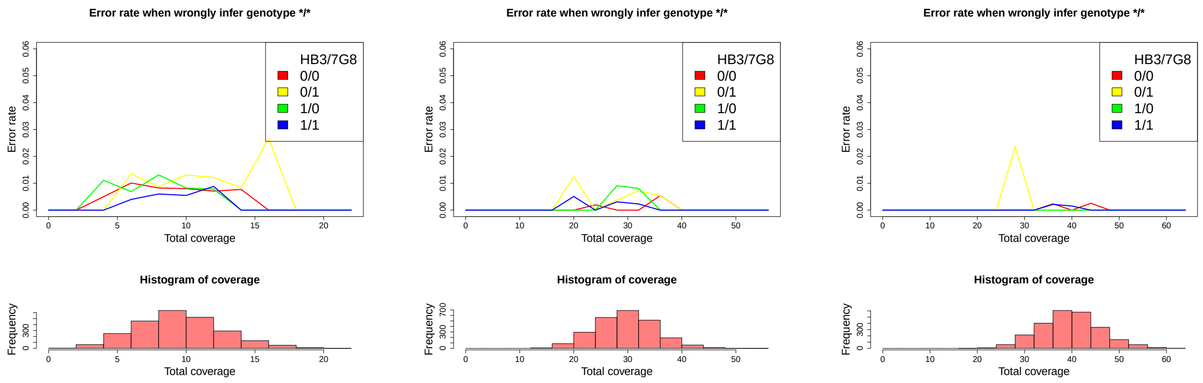

(a) Mean coverage at 10x. (b) Mean coverage at 30x. (c) Mean coverage at 40x.

Figure S3.1: Error rates for inferred genotypes at different read depths.

S4 Comparison to existing methods

In order to show how our method can significantly advance studies in relation to mixed infections, we have compared our inference results on number of strains, strain proportions and haplotypes with `COIL` (Galinsky et al., 2015), `pfmix` (O'Brien et al., 2016), `BEAGLE` (Browning and Browning, 2007) and `SHAPEIT` (Delaneau et al., 2012), using the 27 *in vitro* samples. Code and data are available online at `https://github.com/shajoezhu/DEploid-Supplementary-Materials/tree/master/benchMark`. Note that genotype information is required for benchmarking against `COIL` and `SHAPEIT`, which are inferred by GATK Best Practices(McKenna et al., 2010).

S4.1 `COIL`

`COIL` uses genotype information to infer number of strains. The program input is a text file. Each row contains the sample name, and its sequence, which takes homozygous reference alleles and encodes heterozygous site as "N", and missing site as "X". We used a `python` script to extract reference and alternative allele information from the Pf3k VCF file, and encoded '0/0' as the reference allele, '1/1' as the alternative allele, '0/1' as "N" and './.' as "X". We then submitted the input file to the online portal `http://portals.broadinstitute.org/infect/malaria/coil//`.

S4.2 `pfmix`

The `pfmix` method uses allele count information to infer strain number and proportions. We extracted reference and alternative allele counts, and saved them in text files, which consist of three columns: chromosome information, position, and allele counts. The method works sample-by-sample, inferring the number of strains and proportions based on the allele frequency imbalance within sample. In theory, the method infers strain proportions for between one and eight strains, then uses the Bayesian information criterion to choose the best model. However, we could not manage to get the model selection component of the method to work properly, so we evaluated `pfmix` by fixing he number of strains to the correct value and estimating only the strain proportions.

S4.3 `BEAGLE`

The software package `BEAGLE` input is a VCF file that contains the genotype likelihood information across all 27 samples. We expressed the genotype likelihood by $r\log_{10}(1-x)+a\log_{10}(x)$, where r and a denote the reference and alternative allele counts respectively. Let x denote the read error rate, which takes value of 0.01, 0.5 and 0.99, in the cases of homozygous reference, heterozygous and homozygous alternative respectively. We first converted allele count information into genotype likelihoods using the formula above, then used

`BEAGLE` to infer the haplotypes. We used a `python` script to manipulate the Pf3k VCF file: (1) replacing genotype information '`GT`' inferred by GATK as missing value '0/0's; (2) replacing allele frequencies and read depth by the genotype likelihood '`GL`'. Note that we did not use any additional reference haplotypes for this analysis.

S4.4 `SHAPEIT`

The program `SHAPEIT` takes the genotype information of all 27 samples, and phases the haplotypes chromosome-by-chromosome. The input VCF file must include attribute `GT` in the `FORMAT` field. We split the VCF file by chromosome IDs then used `SHAPEIT` to phase the haplotypes, which are saved in plain text files. We then concatenated the output files, and extracted the haplotypes in each sample. Note that we did not use any additional reference haplotypes for this analysis.

References

Browning, S. R. and B. L. Browning (2007) Rapid and accurate haplotype phasing and missing-data inference for whole-genome association studies by use of localised haplotype clustering. *Am. J. Hum. Genet. 81*(5), 1084–1097.

Delaneau, O. *et al.* (2012) A linear complexity phasing method for thousands of genomes. *Nat. Methods 9*(2), 179–181.

Galinsky, K. *et al.* (2015) COIL: a methodology for evaluating malarial complexity of infection using likelihood from single nucleotide polymorphism data. *Malar. J. 14*(4), 1–9.

Li, N. and M. Stephens (2003) Modeling linkage disequilibrium and identifying recombination hotspots using single-nucleotide polymorphism data. *Genetics 165*(4), 2213–2233.

McKenna, A. et al. (2010). The Genome Analysis Toolkit: a MapReduce framework for analyzing next-generation DNA sequencing data. *Genome Res. 20*, 1297–1303.

O'Brien D,J. *et al.* (2016) Inferring Strain Mixture within Clinical *Plasmodium falciparum* Isolates from Genomic Sequence Data. *PLoS Comput. Biol. 12*(6): e1004824.

Wendler, J. (2015) *Accessing complex genomic variation in* Plasmodium falciparum *natural infection*. Ph. D. thesis, University of Oxford.
